# A Study Protocol of Micro-Ablative Fractional CO2 Laser in Postmenopausal Women With Overactive Bladder Syndrome

**DOI:** 10.7759/cureus.48645

**Published:** 2023-11-11

**Authors:** Konstantinos Kypriotis, Dimitris Zacharakis, Nikolaos Kathopoulis, Michail Diakosavvas, Christos Kalantzis, Anastasia Prodromidou, Stavros Athanasiou, Themos Grigoriadis

**Affiliations:** 1 Obstetrics and Gynaecology, Minimally Invasive Unit, 1st Department of Obstetrics & Gynaecology, National and Kapodistrian University of Athens, Alexandra Hospital, Athens, GRC; 2 Obstetrics and Gynaecology, Urogynaecology Unit, 1st Department of Obstetrics & Gynaecology, National and Kapodistrian University of Athens, Alexandra Hospital, Athens, GRC

**Keywords:** mirabegron, urinary urgency, post-menopause, co2 fractional laser, overactive bladder

## Abstract

Introduction: This is a presentation of a study protocol in order to evaluate whether the application of CO2 laser can additionally benefit the improvement of the symptoms of overactive bladder in postmenopausal women who have just started mirabegron as a treatment.

Materials and methods: This is a study protocol of a randomized double-blind placebo-controlled trial. A total of 50 menopausal women will participate in the study. All patients will start treatment with mirabegron 50 mg and will be randomized into two groups. Women in group A (control) will undergo CO2 laser treatments while those in group B (placebo group) will receive placebo CO2 laser treatments. In total, three monthly sessions will be held in both groups. The monitoring and evaluation of the results will be carried out by completing a three-day urination diary, as well as by completing the Female Lower Urinary Tract Symptoms, Overactive Bladder Questionnaire, King’s Health Questionnaire, Urinary Distress Inventory, Pelvic Floor Impact Questionnaire, Patient Global Impression of Improvement, before each session and a month after the last one. Differences between groups will be assessed at baseline and every month following the three laser therapies.

Results: This is an ongoing study protocol, and we are expecting the analysis of the results in 2024.

Conclusions: The use of laser CO2 in postmenopausal women with overactive bladder syndrome may be a well-tolerated alternative treatment. The goal of our study is to evaluate the efficacy of laser treatment in combination with b3-adrenoreceptor agonist therapy.

## Introduction

According to the International Continence Society (ICS), overactive bladder syndrome (OAB) is defined as urinary urgency with or without urinary incontinence, usually accompanied by frequency and nocturia [1]. OAB is a syndrome that can affect women of various ages and can have a negative impact in their social and sexual life.

The prevalence of OAB in women reaches 30-40% in Europe and USA and increases with age [2]. The age-dependent impact on OAB can be correlated with hypoestrogenism [3] as estrogens are acting on the receptors of the vagina, vulva, urethra and the bladder trigone, in order to maintain the collagen of the epithelium, moisturize the epithelial surfaces and maintain an adequate blood flow.

Initially OAB treatment includes conservative measures of pelvic floor muscle exercises, lifestyle, and behavioral modifications [4]. When these treatments are ineffective, initiation of pharmacological treatment is suggested. There are two classes of drug treatment, the antimuscarinics and the b3-adrenoreceptor agonists. The initial choice of medication therapy is based mostly on the side effects [5]. The antimuscarinics act on the muscarinic receptors and have possible adverse effects on the central nervous system (mouth dryness, constipation). B3-adrenoreceptor agonists act on the selective beta receptors of the detrusor muscle in order to enhance smooth muscle relaxation, presenting similar efficacy with the antimuscarinics with better tolerability.

Recently the use of fractioned CO2 laser seems to present an alternative option of a rapid, safe, and well tolerated treatment for OAB [6-8]. Although there is just a little evidence that can confirm the use of laser in patients with OAB, we presume that it may improve OAB symptoms by decreasing the urogenital age issues, since it seems that the regeneration effect involves all the layers of the vaginal wall, compared with estrogens that are acting superficially [9].

The aim of this study is to add more data to the scientific society by assessing the clinical efficacy and symptom relief of a combination therapy with b3-adrenoreceptor agonist (mirabegron 50mg) and vaginal fractional CO2 laser in post-menopausal women with OAB.

## Materials and methods

The study will be performed in an outpatient urogynecological unit of a tertiary referral center. Institutional Ethical Board was obtained for the present study (N.303) from 1st Department of Obstetrics & Gynecology, National and Kapodistrian University of Athens, Alexandra Hospital, Athens, and its protocol has been registered on ClinicalTrials.gov (NCT03846895). The Declaration of Helsinki was taken into consideration and all procedures comply with the EU General Data Protection Regulation. Potential modifications will be reviewed by the Institutional Ethics Committee and approvals will be obtained. Recruitment of participants will be performed in the urogynecology and the general gynecology outpatient departments. Screening, patient recruitment, and discussion of the study’s objectives and rationale, and obtainment of the signed informed consent forms will be performed by an independent to the study’s design physician. The study’s protocol is in accordance with the Standard Protocol Items: Recommendations for Interventional Trials (SPIRIT). The results of the study will be reported considering the Consolidated Standards of Reporting Trials (CONSORT) statement.

Study design

Initial assessment will include women in menopause who have been experiencing symptoms of overactive bladder. In order to evaluate the participation, patients have to fill in the International Consultation on Incontinence Questionnaire for the evaluation of the Female Lower Urinary Tract (ICIQ-FLUTS).

Symptoms (ICIQ-FLUTS) [10-11], a baseline demographic data, medical history and a three-day micturition diary, presenting the number of voids/incontinence, the volume of every micturition and the frequency of urge urination per day/24h. OAB will be defined by the void frequency and the episodes of urgent urination.

If the woman is eligible to participate, she will be asked to complete a three-day micturition diary for the previous week and fill in the Overactive Bladder Questionnaire (OABq), King’s Health Questionnaire (KHQ), Urinary Distress Inventory (UDI-6), and Pelvic Floor Impact Questionnaire (PFIQ) before every session. The Patient Global Impression of Improvement (PGI-I) questionnaire will be filled in only one time at the end of the study. All information will be evaluated in order to assess the improvement of frequency in micturition, urgent episodes and urinary incontinence, the impact of OAB symptoms on their quality of life, such as daily activities, relationships, feelings, botherness, personal and physical limitations and finally will be evaluated the patient’s impression of improvement one month after the last session in both groups.

The patients enrolled in the study must fulfill the following criteria shown in Table 1. Patients who cannot participate in the study are shown in Table 2.

**Table 1:** Inclusion criteria FSH: follicle stimulating hormone

Inclusion Criteria
Postmenopausal women (≥12 months of amenorrhea or FSH ≥30-40 after total hysterectomy)
Overactive bladder syndrome: ≥three months symptoms of urgency, with or without urinary incontinence, and ≥eight episodes/24h
At least three episodes of urgent urination (3rd-4th grade) under PPiUS (patient perception intensity of urgency scale) during a three-day urination calendar, with or without urinary incontinence

**Table 2:** Exclusion Criteria

Exclusion Criteria
Genital prolapse >2nd degree according to the Pelvic Organ Prolapse classification system
Voiding residue volume >200 ml
Obstruction bladder
Use of vaginal estrogen in the last month
Use of drugs for urinary incontinence
Use of psychiatric drugs
Urinary or genital infection
Kidney or liver disease
Cardiac conduction or rate disorders
Arterial hypertension unregulated
Neurological diseases and diabetic neuropathy
Myasthenia
Cancer
Previous radio-chemotherapy

Randomization

Eligible patients are randomized 1:1 using a computer-generated randomization scheme, to receive active laser therapy or placebo, once monthly for three months.

Interventions

All participants will receive treatment with mirabegron 50mg and they will equally be divided in active or placebo laser therapy. Fractional CO2 laser (SmartXide2 V2LR, Monalisa Touch, DEKA, Florence, Italy) will be applied at monthly intervals in both groups. The parameters of the machine can be changed manually and are presented in Table 3.

**Table 3:** The machine laser parameters that are going to be used in each session of each group.

Laser Group	Placebo Group
Microablative fractional CO2 laser therapy at monthly intervals	Placebo CO2 laser therapies at monthly intervals
Power: 40 watts	Power: 0.5 watts
Dwell time: 1000μs	Dwell time: 1000μs
Spacing 1000 μm	Spacing 1000 μm
Depth: SmartStak parameter 3	Dwell time: 1000μs
D-pulse mode	Smart-pulse mode

Both groups will receive a total of three sessions. The technique of the vaginal therapy will be identical for both groups. The basic difference in parameters of these two groups are in power and emission mode.

Blinding

The investigator, study site personnel and patients will all be blinded to treatment. An unblinded nurse will be responsible for programming the laser parameters, before the doctor and patient enter the examination room. The screen of the laser system will be covered to ensure proper blinding. Although in the placebo group the power of 0.5 watts and smart pulse emission mode will not allow energy submission to the vaginal tissue, the identical laser sound and treatment technique as in the active group will cover the placebo procedure.

Data collection methods

The study consists of four clinical visits (months 0, 1, 2, 3). Demographics and other baseline characteristics will be collected at the start of run-in period (month 0). Patients will complete the questionnaires mentioned (OABq, KHQ, UDI-6, PFIQ) in every visit and a three-day micturition diary a week before it. In addition, the Patient Global Impression of Improvement questionnaire (PGI-I) will be added in the last visit. Patients are advised to record twice a day their blood pressure and pulse rate, a week before the randomization and at every visit.

Primary Outcomes

The primary outcomes will be assessed by the score of the OAB-q questionnaire that consists of an eihjt-item symptom bother scale section and a 25-item health-related quality of life scale section and by the completion of a three-day voiding diary, monitoring the frequency of micturition, the urgency, and the urinary incontinence.

Secondary Outcomes

The secondary outcomes will be assessed by the scores of the following validated questionnaires: 1) King’s Health Questionnaire (KHQ); 2) Urinary Distress Inventory (UDI-6); 3) Pelvic Floor Impact Questionnaire (PFIQ-7); 4) Patient Global Impression of Improvement (PGI-I).

Sample Size

Power analysis methodology represents a design, with two levels of the between-subject factor of two study groups and four levels of the within-subjects factor of time. A repeated measures ANOVA power analysis was conducted. The effect size for this calculation used the ratio of the standard deviation of the effects for a particular factor or interaction and the standard deviation of within-subject effects. The power analysis was conducted for a single, two-group between-subjects factor, and a single within-subjects factor assessed over four time points. For this design, 50 participants (25 per group) achieve a power of 0.80 for the within-subjects main effect at an effect size of 0.25; a power of 0.80 for the between-subjects main effect at an effect size of 0.32 and a power of 0.80 for the interaction effect at an effect size of 0.25.

Statistical analysis

Comparison of data between the compared groups in both phases will be based on the intention-to-treat principle. Randomisation will be done for all women. Later dropouts and those not adhering to protocol will be excluded. The normality assumption will be evaluated using the Kolmogorov-Smirnov test. Student’s t-tests will be computed for the comparison of a continuous variable between two groups when the distribution is normal and the Mann-Whitney test when the distribution is not normal. Paired t-tests and Wilcoxon signed rank tests will be used for pre and post comparisons. 

For the comparison of proportions between the two study groups, Chi-square and Fisher’s exact tests will be used. Repeated measurements ANOVA will be performed to evaluate the changes observed between control and intervention group over the follow-up period. Additionally, three sensitivity analyses will be performed: per-protocol analysis, robustness of trial accounting for missing data, and adjusting for baseline covariates. Per-protocol analysis will include women who will complete the therapeutic protocol.

Adjustment for confounding factors will be performed using mixed linear regression models that account for multiple measurements per individual obtained at different time points. All reported p values will be two-tailed and statistical significance is set at p<0.05.

Adverse effects

Patients with severe or uncontrolled hypertension should not take mirabegron and will be excluded from the study. While clinical trials have not demonstrated significant increases in blood pressure compared with placebo [12], patients can develop hypertension. Blood pressure will be monitored, and in case of any adverse event, blood pressure medications could be adjusted according to the primary care clinician's suggestion.

## Results

This study evaluates the use of a CO2 laser (in combination with mirabegron) to treat symptoms of overactive bladder in postmenopausal women in a randomized, double-blind, placebo-controlled study. This is an ongoing study protocol and the results are going to be collected and analysed in early 2024.

## Discussion

Currently there is a wide range of therapeutic options for the treatment of overactive bladder syndrome. However, complete remission is not often achieved. The need for more effective treatment has endorsed the use of combined treatment modalities. Several studies have been published showing the efficacy of a combination therapy.

In a multicenter randomized controlled trial, the combination of pelvic floor exercises with behavioral modifications and anticholinergic therapy, showed better results on bladder symptoms than with conservative treatment only [13].

Two trials comparing the use of mirabegron plus solifenacin and solifenacin alone showed that combination therapy had better results in OAB symptoms than monotherapy [14,15].

Combination therapy of anticholinergics with topical estrogen also seems to have better results than monotherapy. Two studies comparing the use of monotherapy with antimuscarinics (fesoterodine, solifenacin) and combined therapy with topical estrogens (Premarin, promestriene) showed better results for the combination groups with improvement in OAB symptoms [16,17].

Recently the use of fractional microablative CO2 and Er:YAG laser seems to have a role in the treatment of OAB in postmenopausal women, since the laser thermal effects have benefits to the urinary tract by inducing neocollagenesis, collagen remodeling and elastin stimulation, giving promising results for the treatment of OAB in postmenopausal women.

Aguiar et al. conducted a randomized trial of 72 postmenopausal women with urinary symptoms divided into three groups: receiving fractional CO2 laser or vaginal lubrification or vaginal estrogen therapy. The laser group was the only group that showed reduction of the scores in frequency and interference of urinary loss in daily life from the ICIQ-UI SF questionnaire and in comparison, with the ICIQ-OAB scores, showing significantly better results and statistically significant reduction of nocturia after treatment.

Perino et al. conducted a pilot study of 30 post-menopausal women with vulvovaginal atrophy (VVA) and OAB symptoms, treated with a total of three sessions of fractional CO2 laser every 30 days. The results showed an improvement in VVA symptoms, in number of urge episodes and in the OAB-q score.

Finally, another study was described by Okui, who examined the efficacy of non-ablative vaginal erbium: YAG laser (VEL) in post-menopausal women with OAB syndrome. One hundred fifty patients were enrolled and divided into three groups, receiving treatment with an anticholinergic or a β3-adrenoreceptor agonist 25mg or VEL. The results showed improvement in OAB score in all groups at 12 months after the initiation of the therapy [8].

In all of these studies there is a lack of either a placebo-controlled arm or long-term follow-up or larger sample size, failing to provide strong scientific evidence for the use of laser in OAB syndrome. 

## Conclusions

Overactive bladder syndrome remains a common, challenging and stressful symptom. Its pathophysiology is still not being well comprehended. Pharmacological treatment seems to remain an important option and in combination with pelvic floor muscle exercises and behavioral modifications can present even better results. Since lately the use of laser in women with stress urinary incontinence and genitourinary syndrome of menopause presents an alternative option therapy, it makes us assume that it might also have a role in overactive bladder symptoms. At the end of this study we aim to provide valid data in order to evaluate the use of fractional CO2 laser as a co-therapy with mirabegron in post-menopausal women with overactive bladder syndrome.
